# Fu Fang Zhen Zhu Tiao Zhi Capsules Protect against Myocardial Ischemia by Inhibiting Cardiomyocyte Pyroptosis

**DOI:** 10.1155/2022/4752360

**Published:** 2022-11-02

**Authors:** Xiaoqi Shao, Bingying Huang, Huiling Tan, Ruonan Wang, Xueying Huang, Hongtao Diao, Jiawen Cheng, Mengxian Sun, Dongwei Wang, Kaili Wu, Meiling Yan, Xianglu Rong, Yue Zhang, Jiao Guo

**Affiliations:** ^1^Guangdong Metabolic Diseases Research Center of Integrated Chinese and Western Medicine, Guangzhou 510006, China; ^2^Key Laboratory of Glucolipid Metabolic Disorder, Ministry of Education of China, Beijing, China; ^3^Institute of Chinese Medicine, Guangdong Pharmaceutical University, Guangzhou 510006, China; ^4^Guangdong TCM Key Laboratory for Metabolic Diseases, Guangzhou 510006, China

## Abstract

**Background:**

Fu Fang Zhen Zhu Tiao Zhi (FTZ) is a traditional Chinese herbal prescription widely used to treat dyslipidemia, metabolic diseases, and diabetic coronary disorders. Cardiomyocyte death and loss of regenerative ability cause cardiac dysfunction and heart failure. FTZ can effectively treat diabetic cardiomyopathy and macrovascular diseases; however, the mechanism behind the phenomenon is still unclear. Here, we determined the mechanism of action of FTZ in treating myocardial infarction.

**Methods:**

Male C57BL/6 mice were treated with 2.4 or 1.2 g/kg FTZ, or administered saline by oral gavage daily for four weeks, and a 24-hour ligation was administered to the artery. Echocardiography was used to evaluate cardiac function. Hematoxylin and eosin and Evans blue/triphenyltetrazolium chloride staining were carried out by staining the cardiac tissue, used to evaluate cardiac function and infarct size. Using western blotting and reverse transcriptase-polymerase chain reaction, we determined the relative levels of NOD-like receptor protein (NLRP) 3, ASC, cleaved caspase-l (C-Caspase-1), GSDMD, and GSDMD-N. TUNEL, immunohistochemical, and immunofluorescence staining were used to determine cell death and NLRP3 expression. An enzyme-linked immunosorbent assay (ELISA) was used to detect the levels of interleukin (IL)-1*β* and IL-18.

**Results:**

FTZ reduced ischemia-induced cardiomyocyte cell death *in vivo* and H_2_O_2_-induced cell death *in vitro* by maintaining cardiac architecture and restoring cardiac function. FTZ decreased the NLRP3 expression and inhibited pyroptosis-correlated genes, including NLRP3, ASC, GSDMD, C-Caspase-1, and GSDMD-N. NLRP3 overexpression impaired the efficacy of FTZ by inducing pyroptosis.

**Conclusion:**

FTZ could preserve cardiac function resulting from ischemic insult by inhibiting pyroptosis, which was partially reversed by NLRP3 overexpression, indicating that NLRP3 could be a potential target of FTZ in treating myocardial infarction.

## 1. Introduction

Myocardial infarction (MI) represents a chief cause of chronic heart failure worldwide [1]. Cardiac remodeling after MI results in heart failure, which is responsible for an increase in socioeconomic burden [2]. Finding a lasting and effective agent for the management of these patients is, therefore, a valid approach.

Fu Fang Zhen Zhu Tiao Zhi (FTZ) is a traditional Chinese herbal medicine consists mostly of eight types of herbs [3] and used to treat coronary heart disease [4], nonalcoholic steatohepatitis [5], atherosclerosis [6], diabetes [5], and aging [7, 8]. FTZ has been shown to benefit multiple organs and has been used to treat cardiac diseases. We recently discovered that FTZ inhibits inflammation and cardiac fibrosis and ameliorates diabetic cardiomyopathy in mice. Moreover, favorable results have been reported in a clinical trial on FTZ [9, 10]. However, the mechanism of action of FTZ in treating cardiac disorders remains unclear.

There are multiple mechanisms of cardiomyocyte death after MI, including apoptosis, necrosis, pyroptosis, autophagy, and ferroptosis. A difference between apoptosis and pyroptosis is that pyroptosis progresses via the involvement of the inflammasome and gasdermin family, whereas apoptosis does not [11]. Caspases 1, 4, 5, and 11 can drive pyroptosis, resulting in large pores [12, 13]. Simultaneously, the broken cells release proinflammatory cytokines such as interleukin (IL)-1*β*, IL-18, and other cellular contents. Among the inflammasome proteins, NOD-like receptor protein (NLRP) 3 is well characterized in cardiac ischemia. NLRP3 activates pyroptosis and further aggravates cardiac function by releasing proinflammatory cytokines [14, 15]. Several studies have demonstrated that inhibiting NLRP3 preserves functions of the heart, both systolic and diastolic by pyroptotic impairment [16].

Herein, we used multiple methods to determine the efficacy of FTZ in ischemic cardiac disorders and found that FTZ could improve myocardial structure and function by preventing pyroptosis. Thus, this study could broaden our understanding of FTZ in cardiac ischemia.

## 2. Materials and Methods

### 2.1. Preparation of FTZ Extract

FTZ is composed of Citri Sarcodactylis Fructus, Fructus Ligustri Lucidi, Salviae Miltiorrhizae Radix et Rhizoma, Notoginseng Radix et Rhizoma, Coptidis Rhizoma, Atractylodis Macrocephalae Rhizoma, Cirsii Japonici Herba et Radix, and Eucommiae Cortex. The herbal formula of FTZ is listed in Table 1. The plant material was authenticated by Professor Wei He of Guangdong Pharmaceutical University based on the pharmacopeia of the People's Republic of China identification key (ISBN 2015, volume I), and the voucher specimens were labeled GDPUZYY 20110901–8 [17]. FTZ was provided by Guangdong Pharmaceutical University. High-performance liquid chromatography fingerprint analysis was conducted to verify the quality of the FTZ extract (Figure S1) [18].

Plant names were checked using the resource http://www.theplantlist.org [19].

### 2.2. Ethics Statement

C57BL/6 adult male mice (∼8 weeks old, weighing 20 ± 2 g) were purchased from Liaoning Changsheng Biotechnology Corporation, and Guangdong Medical Laboratory Animal Center provided neonatal mice for this study. A temperature-controlled room at 25 ± 2°C was provided with standard solid food and water throughout the experiment. All procedures were approved by the Laboratory Animal Care and Use Committee at Guangdong Pharmaceutical University (permit number: Gdpulacspf2017277).

### 2.3. Mouse Model of MI and Drug Administration

The mice were randomly divided into three equal groups: the sham group, the MI group, and the MI + FTZ (1.2 mg·kg^−1^) group. In the treatment groups, mice were administered an oral medication of FTZ at a dose of 1.2 mg·kg^−1^ daily for 2 days, whereas those in the other groups, an equivalent volume of solvent was given for 28 days prior to the surgery to induce MI. Mice were anesthetized and then intubated to a rodent ventilator (Shinano, Tokyo, Japan). There was a small incision made at the third and fourth intercostal spaces of the left thoracotomy, and a 7–0 prolene suture was applied to ligate the left anterior descending (LAD) coronary artery. Approximately 24 h after MI, mice were sacrificed by euthanasia. Randomization and blinding were adopted for all animal experiments. Surgical procedures for the sham control mice were identical to those used to establish the mouse model of MI, but without the coronary artery ligation step.

### 2.4. Echocardiography

Before surgery and after 24 h of MI, mice were anesthetized and subjected to echocardiography, using a Vevo 2100 ultrasound machine for echocardiography (Visual Sonics, Toronto, Canada) at a probe frequency of 30 MHz. At the papillary muscles, two-dimensional images were obtained. The instrument calculated the average of three cardiac cycles for ejection fraction (EF) and fractional shortening (FS) based on M-mode tracings.

### 2.5. Measurement of Myocardial Infarct Size

Mice were injected with Evans's blue (1%; Solarbio, China) through the abdominal aorta to stain the noninfarcted areas. Hearts were cut into 1 mm-thick sections and stained for 15 min with 2% triphenyltetrazolium chloride (TTC; Solarbio) at 37°C. An optical microscope was used to capture the images (ZEISS, Germany).

### 2.6. Primary Culture of Neonatal Mice Ventricular Cardiomyocytes (NMVCs)

One- to three-day-old neonatal mice were subjected to open-chest surgery to expose the hearts. Heart tissues were digested with 0.25% trypsin (Solarbio, China) to yield single cardiomyocytes. Heart tissues were trypsinized until tissues disappeared, and the cell suspensions were collected by centrifugation at 1000 rpm for 5 min. Isolated cells were resuspended in DMEM (Hyclone, Logan, UT, USA) containing 10% fetal bovine serum (Hyclone, Logan, UT, USA) and penicillin/streptomycin (100 U/mL; Beyotime, Shanghai, China). Cardiomyocytes were purified by centrifuging for different time intervals. The resuspension was plated onto a culture flask for 120 min at 37°C, allowing for the preferential attachment of fibroblasts to the bottom. Cardiomyocytes were removed and seeded into plates, incubated at 37°C with 5% CO_2_ and 95% air in a humidified incubator, and then treatments were administered. In hypoxia treatment, cardiomyocytes were exposed to H_2_O_2_ for 24 h, whereas for FTZ interventions, cardiomyocytes were treated with 100 nmol/mL FTZ for 24 h.

### 2.7. Isolation and Culture of Neonatal Mouse CM

The primary cardiomyocytes of neonatal mice mainly include cardiomyocytes, fibroblasts, and endothelial cell. On the first day, whole hearts were removed from the mice and washed with precooled 10% FBS and 1% penicillin/streptomycin for three times. Then, discard the liquid and add 3 ml PBS and 2 ml trypsin, put it into a 50 ml centrifuge tube, and shake it at 4°C for 8–12 h. After 8–12 h, add 5 ml of DMEM complete medium to the 50 ml centrifuge tube containing the heart to neutralize the trypsin. The tissues were digested with type II collagenase. The cells were pelleted by centrifugation at 1000 rpm for 5 min. The cell pellets were resuspended in Dulbecco's modified Eagle's medium with 10% FBS and 1% penicillin/streptomycin, plated onto a T25 flask and incubated at 37°C for least 2 h.

### 2.8. Cell Transfection

NLRP3-overexpressing pcDNA3.1 plasmid (100 nM) or NCs (empty pcDNA3.1 plasmid) were synthesized and transfected into neonatal cardiomyocytes using Lipofectamine 2000 reagent (Invitrogen, Carlsbad, CA, USA). After 8 h of cell culture, a fresh medium was used for 48 h.

### 2.9. Terminal Deoxynucleotidyl Transferase dUTP Nick End Labeling (TUNEL) Assay

In order to assess drug-induced pyroptosis in cardiomyocytes, 24-well plates were used. The cardiomyocytes were fixed and permeabilized with paraformaldehyde and Triton X − 100 at 4% and 1%, respectively. The cells were incubated with TUNEL staining agents at 37°C in the dark for 1 h and with 4′,6-diamidino-2-phenylindole (DAPI) for 15 min. Fluorescence was determined using confocal laser scanning microscopy (FV300, Olympus, Japan).

### 2.10. Immunohistochemical (IHC) and Immunofluorescence (IF) Assays

Heart tissue sections were soaked in EDTA antigen retrieval buffer (pH 8.0). Then, the sections were incubated with the homologous primary antibody for 30–40 min at 37°C. The negative control group consisted of normal antirabbit immunoglobulin G horseradish peroxidase polymer conjugated with the second antibody to treat the sections for 30 min, after which diaminobenzidine solution was used for IHC analysis. For IF studies, fluorescein bonded with a secondary antibody was used to treat the sections, which were then visualized using fluorescence microscopy.

### 2.11. Hematoxylin and Eosin (H&E) Staining

Heart sections were fixed in 4% paraformaldehyde. Then, the tissue samples were embedded in paraffin and each sample was cut into 5 *μ*m-thick sections. H&E staining was performed to evaluate histopathological alterations. Images were captured using microscopy (Olympus Corporation, Tokyo, Japan).

### 2.12. Enzyme-Linked Immunosorbent Assay (ELISA)

IL-1*β* or IL-18 concentrations in the cell supernatant or serum were determined using an ELISA kit (Elabscience Biotechnology Co., Ltd). In brief, add 100 *μ*L of standard working solution or serum sample to the corresponding plate wells and incubate at 37°C for 90 min. Discarding the liquid, immediately add 100 *μ*L working solution and incubate at 37°C for 60 min. Then, add 100 *μ*L of HRP enzyme conjugate working solution to each well, and incubate at 37°C for 30 min. Finally, add 90 *μ*L of substrate solution and incubate at 37°C for about 15 min, stop the reaction and immediately read at 450 nm wavelength and process the data.

### 2.13. Lactate Dehydrogenase (LDH) Release Assay

In the cell-free supernatant, LDH levels reflect cellular permeability. LDH was measured with an assay kit at 490 nm from the cell-free supernatant (Beyotime). In brief, inoculate appropriate cells into a 96-well cell culture plate according to the size and growth rate of the cells. After H_2_O_2_ stimulation, the cell culture plate was centrifuged at 400 g for 5 min in a multiwell plate centrifuge. Aspirate the supernatant, add 150 *μ*L of the LDH release reagent with PBS, and shake the culture plate in the cell incubator. Then, the cell culture plate was centrifuged at 400 g for 5 min with a multiwell plate centrifuge. Added 120 *μ*L of the supernatant to a new 96-well culture plate. Finally, add 60 *μ*L LDH detection working solution to each well, put it at room temperature in the dark for 30 min and measure absorbance at 490 nm.

### 2.14. Western Blotting

Neonatal cardiomyocytes cells were collected, digested with trypsin, and washed twice with phosphate-buffered saline after treatment with H_2_O_2_ or FTZ. A lysis buffer containing 1% protease inhibitor is used to extract proteins from the samples (Roche, Switzerland). Protein concentrations were measured using a bicinchoninic acid protein kit (Beyotime Institute of Biotechnology, Shanghai, China). The proteins were separated using 12% sodium dodecyl sulfate-polyacrylamide gel electrophoresis and transferred to nitrocellulose membranes (PALL, USA). Membranes were incubated with antibodies for 50 min in the dark. Each protein band was quantified using an infrared fluorescence imaging detector (LI-COR Bioscience) and normalized to *β*-actin.

### 2.15. RNA Isolation and Real-Time PCR

TRIzol reagent was used to extract RNA from myocardial tissues or NMVCs (Invitrogen, Carlsbad, CA, USA) according to the manufacturer's instructions. A total of 1 *μ*g of RNA was reverse-transcribed into complementary DNA under the conditions of 37°C for 15 min, 98°C for 5 min, and 4°C using a reverse-transcription kit (Toyobo, Osaka, Japan). SYBR Green (Toyobo) was used for real-time PCR to quantify messenger RNA (mRNA) levels using a 7500 Fast Real-Time PCR System (Biosystems, Singapore). *β*-actin was used as an internal control. Gene expression was measured using the 2^−ΔΔCT^ method. Primer sequences are listed in Table 2.

### 2.16. Statistical Analysis

The data are presented as the mean ± standard error of the mean. Student's *t*-test was used for comparisons of the two groups and a one-way analysis of variance was followed by Tukey's postcorrection for comparisons of multiple groups. *p* < 0.05 was considered to indicate a significant difference. GraphPad Prism 5 was used for statistical analysis.

## 3. Results

### 3.1. FTZ Improves Cardiac Function after MI in Mice

**Figure 1:**
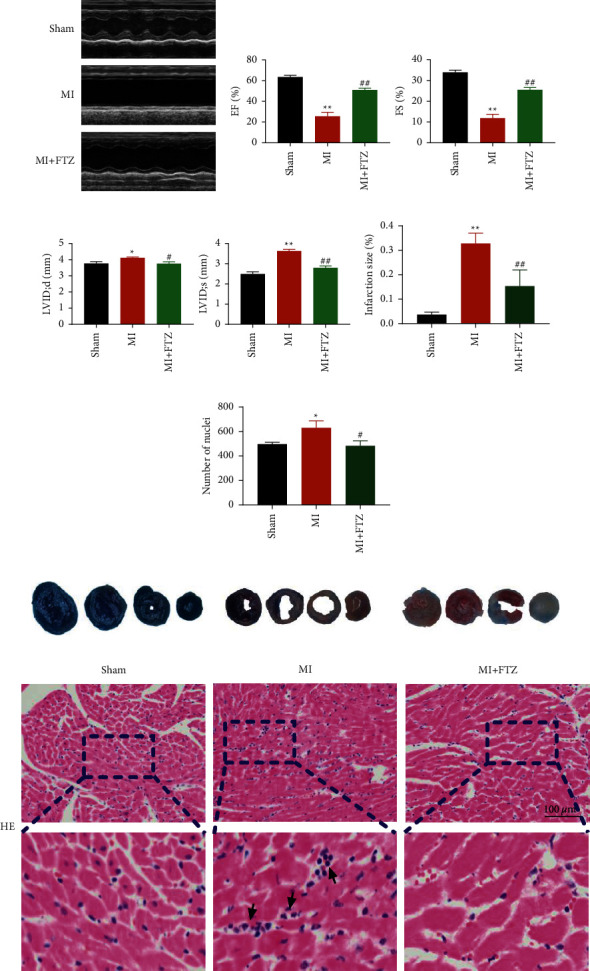
FTZ improves dysregulation in the cardiac function and structure in mice with MI. (a–c) Echocardiograms and EF%, FS%, LVIDd, and LVIDs in the sham, MI, and MI + FTZ groups. ^*∗*^*p* < 0.05, ^*∗∗*^*p* < 0.01 vs. sham; ^#^*p* < 0.05 and ^##^*p* < 0.01 vs. MI; *n* = 8 for EF%, FS%, LVIDd, and LVIDs. (d–e) Quantitative data analysis of TTC-stainedand H&E staining. (f) Representative TTC-stained transverse sections; *n* = 3. (g) H&E staining of the heart tissue after FTZ treatment. Black arrows indicate positions of inflammation infiltrate. Scale bar indicates 100 *μ*m; *n* = 3.

To verify the therapeutic effects of FTZ, we induced MI after FTZ treatment. Echocardiography findings indicated that FTZ significantly increased the EF% and FS% in the MI + FTZ group (Figures 1(a) and 1(b)). Moreover, compared with mice in the MI group, The representative long axis images of the left ventricular internal diameter at the end diastole (LVIDd) and the left ventricular internal diameter at the end systole (LVIDs) of mice treated with FTZ showed a decrease (Figure 1(c)). To determine the expansion of infarct size after myocardial injury, we measured the infarct size in sham and FTZ-treated mice. TTC/Evans blue double staining showed that the infarct size of mice in the MI + FTZ group was dramatically reduced (Figures 1(d) and 1(f)). H&E staining indicated fewer inflammatory cells and compact cardiac structure (Figures 1(e) and 1(g)). These results suggested that FTZ preserved cardiac function after MI.

Fu Fang Zhen Zhu Tiao Zhi (FTZ); myocardial infarction (MI); left ventricular internal diameter at end diastole (LVIDd); left ventricular internal diameter at end systole (LVIDs); triphenyltetrazolium chloride (TTC); and hematoxylin and eosin (H&E).

### 3.2. FTZ Inhibits Pyroptosis in Acute Ischemic Hearts

**Figure 2:**
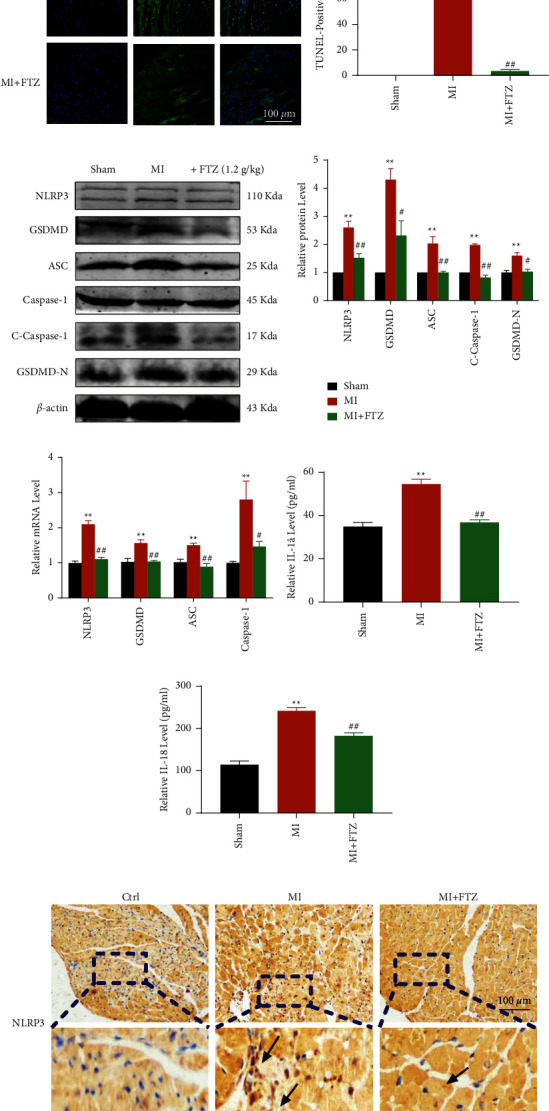
FTZ inhibits pyroptosis in ischemic hearts. (a and b) Double-fl;uorescence staining with DAPI (blue) and TUNEL (green) and quantitative TUNEL data. Scale bar indicates 100 *μ*m (right);*n* = 3. (c and d) Representative western blots (3 independent experiments) (left) and quantiflcation of western blots. *β*-actin was used as an internal control. (e) Quantitative RT-PCR for NLRP3, GSDMD, ASC, C-Caspase-1, 1L-1*β*, and 1L-18. (f and g) Serum IL-18 and IL-1*β* levels were measured using ELISA. ^*∗*^*p* < 0.05, ^*∗∗*^*p* < 0.01 vs. sham; ^#^*p* < 0.05, ^##^*p* < 0.01 vs. MI. (h) NLRP3 representative IHC images. Black arrows indicate the positions of NLRP3. Scale bar indicates 100 *μ*m; *n* = 3.

To further explain the precise treatment effect of FTZ in MI, TUNEL staining was performed, compared to the control group, the MI group had significantly more TUNEL-positive cells and was considerably blunted after FTZ treatment (Figures 2(a) and 2(b)). To identify whether the apoptotic cells are cardiomyocytes, endothelial cells, or other cells and whether the apoptosis cell is NLRP3 positive. We stained the cardiac tissue with TUNEL and a-actinin or NLRP3 and a-actinin. The results showed the apoptosis cell was cardiomyocytes, but unfortunately, NLRP3 positivity is not characteristic, but we believe that pyroptosis occurs primarily in cardiomyocytes (Figures S2(a) and S2(b)). Then, we measured the protein levels of NLRP3, ASC, cleaved caspase-l (C-Caspase-1), and GSDMD-N in ischemic hearts with or without FTZ treatment. As shown in Figures 2(c)–2(e), the pyroptotic markers including NLRP3, ASC, C-Caspase-1, and GSDMD-N, as well as their transcription, were markedly reduced in FTZ-treated mice. Consistently, FTZ decreased IL-18 and IL-1*β* levels in mouse serum (Figures 2(f)–2(g)). Besides, IHC staining confirmed that NLRP3 expression increased in mice in the MI group and could be partially reversed by FTZ (Figure 2(h)), suggesting its role in improving cardiac remodeling and function by inhibiting pyroptosis.

Fu Fang Zhen Zhu Tiao Zhi (FTZ); myocardial infarction (MI); left ventricular internal diameter at end diastole (LVIDd); left ventricular internal diameter at end systole (LVIDs); triphenyltetrazolium chloride (TTC); hematoxylin and eosin (H&E); 4′,6-diamidino-2-phenylindole (DAPI); terminal deoxynucleotidyl transferase dUTP nick end labeling (TUNEL); NOD-like receptor protein (NLRP); reverse transcriptase-polymerase chain reaction (RT-PCR); interleukin (IL); immunohistochemical (IHC); and enzyme-linked immunosorbent assay (ELISA).

### 3.3. FTZ Inhibits Oxidative Stress-Induced Pyroptosis *In Vitro*

**Figure 3:**
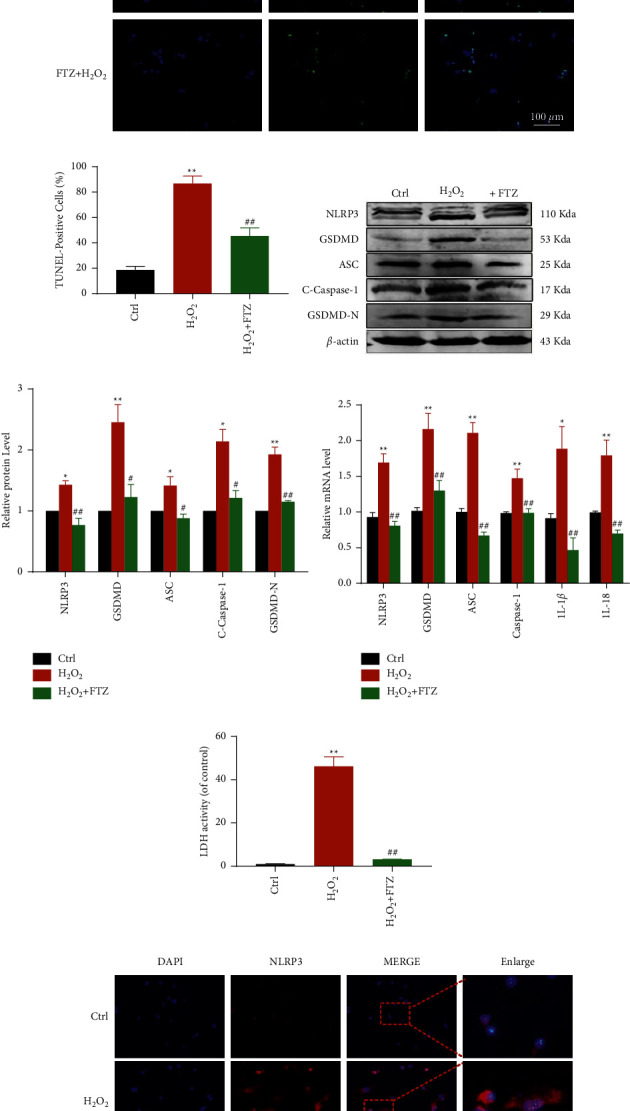
FTZ inhibits pyroptosis *in vitro*. (a and b) Double-fl;uorescence staining with DAPI (blue) and TUNEL (green) and quantitative TUNEL data. Scale bar indicates 100 *μ*m (right); *n* = 3. (c and d) Representative western blots (3 independent experiments) (left) and quantiflcation of western blots in NMCMs. *β*-actin was used as an internal control. (e) Quantitative RT-PCR for NLRP3, GSDMD, ASC, C-Caspase-1, and GSDMD-N. (f) The degree of cell insult was assessed using % LDH release. ^*∗*^*p* < 0.05 and ^*∗∗*^*p* < 0.01 vs. CTRL;^#^*p* < 0.05 and ^##^*p* < 0.01 vs. H_2_O_2_. (g) NLRP3 representative immunofluorescence images. Scale bar indicates 100 *μ*m; *n* = 3.

Then, we measured cell viability at different doses to test the protective effects of FTZ *in vitro* and selected a dose of 50 *μ*g as optimal (Figure S3). Oxidative stress plays a critical role in pyroptosis. TUNEL-positive cells significantly increased in H_2_O_2_-treated cardiomyocytes and were blunted after treatment with FTZ (Figures 3(a) and 3(b)). In order to determine which cell type undergoes apoptosis, we stained DAPI, *α*-actinin, and TUNEL. Results showed that cardiomyocytes undergo the most apoptosis (Figure S4). Moreover, NLRP3, GSDMD, ASC, C-caspase-1, GSDMD-N, and their transcription levels showed a marked decline in the FTZ-treated group (Figures 3(c)–3(e)). The decrease in LDH release after FTZ treatment reflected reduced damage (Figure 3(f)). Inflammasome-activatedN-terminal cleavage product (GSDMD-NT) ruptures membrane pores and induces pyroptosis. Compared with H_2_O_2_ treatment, FTZ treatment inhibited the intensity of the NLRP3 inflammasome (Figure 3(g)), indicating the inhibitory effect of FTZ in oxidative stress-induced pyroptosis.

Fu Fang Zhen Zhu Tiao Zhi (FTZ); myocardial infarction (MI); 4′,6-diamidino-2-phenylindole (DAPI); terminal deoxynucleotidyl transferase dUTP nick end labeling (TUNEL); NOD-like receptor protein (NLRP); reverse transcriptase-polymerase chain reaction (RT-PCR); and lactate dehydrogenase (LDH).

### 3.4. FTZ Reduces Cardiomyocyte Pyroptosis by Inhibiting NLRP3

**Figure 4:**
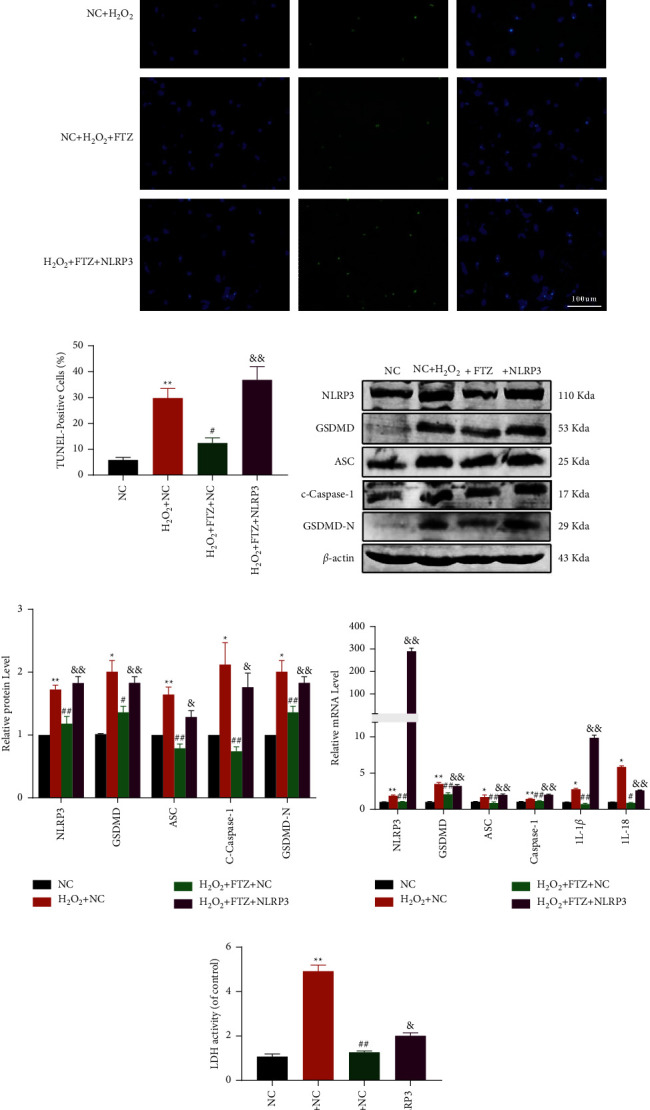
NLRP3 is a target of FTZ. (a and b) Double-fluorescence staining with DAPI (blue) and TUNEL (green) and quantitative TUNEL data. Scale bar indicates 100 *μ*m (right); *n* = 3. (c and d) Representative western blots (3 independent experiments) (left) and quantiflcation of western blots in NMCMs. *β*-actin was used as an internal control. (e) Quantitative RT-PCR for NLRP3, GSDMD, ASC, C-Caspase-1, and GSDMD-N. (f) Degree of cell insult was assessed using % LDH release. ^*∗*^*p* < 0.05 and ^*∗∗*^*p* < 0.01 vs. NC; ^#^*p* < 0.05 and ^##^*p* < 0.01 vs. H_2_O_2_ + NC; and *p* < 0.05 and *p* < 0.01 vs. H_2_O_2_ + FTZ + NC.

Several studies have shown that overactivation of NLRP3 can promote inflammatory infiltrates and inhibit cell viability [20]. To overexpressed NLRP3 in cardiomyocytes to determine whether it was a directed target of FTZ. Therefore, we assessed cardiomyocytes purity and NLRP3 plasmid transfection efficiency in primary cardiomyocytes. The results showed the majority of the isolated cells were cardiomyocytes, and a 0.5 nanomolar concentration was optimal for transfection (Figures S5(a) and S5(b)). The TUNEL staining demonstrated that FTZ treatment could decrease cell pyroptosis (Figures 4(a) and 4(b)). FTZ treatment led to a decrease in the expression and transcription of NLRP3, C-caspase-1, GSDMD, ASC, and GSDMD-N (Figures 4(c)–4(e)), which was impaired by NLRP3 overexpression. NLRP3 overexpression also increased LDH levels *in vitro* (Figure 4(f)). Despite this, too many variables can contribute to myocardial infarction. So, we tried to specifically inhibit its expression by its specific inhibitor MCC950 in cardiomyocytes and access the viability. The results showed both FTZ and MCC950 could improve cell viability (Figure S6). These results suggested that FTZ could protect cardiomyocytes via the NLRP3 inflammasome pathway.

Fu Fang Zhen Zhu Tiao Zhi (FTZ); 4′,6-diamidino-2-phenylindole (DAPI); terminal deoxynucleotidyl transferase dUTP nick end labeling (TUNEL); NOD-like receptor protein (NLRP); reverse transcriptase-polymerase chain reaction (RT-PCR); and lactate dehydrogenase (LDH).

## 4. Discussion

In this study, we examined the effects of FTZ on ischemic heart diseases and investigated its underlying mechanisms. FTZ reduces the infarct size and exerts a protective effect on the heart. Most importantly, FTZ significantly reduced the expression of NLRP3, ASC, and caspase-1*in vivo* and *in vitro*. Moreover, FTZ suppressed the proinflammatory cytokines including IL-1*β* and IL-18, reduced pyroptotic cell death, and preserved cardiac function. Our results indicated that FTZ might improve the contractile function by affecting both the NLRP3 inflammasome and pyroptosis.

Acute cardiac injury after MI remains a leading cause of mortality and disability [21]. Long-term ischemia induces an inevitable loss of cardiomyocytes, which further exacerbates cardiac function [22, 23]. Previous studies have noted the importance of FTZ in cardiovascular diseases [4, 24]. This compound contains multiple active ingredients and has been clinically tested for the treatment of metabolic diseases linked to glucose and lipids. It has been reported that FTZ modulates NLRP3 inflammasome formation and activation and protects against diabetes-induced coronary damage [25]. FTZ significantly suppressed the production of several pyroptosis-related proteins in our study, including NLRP3, ASC, GSDMD-N, and C-Caspase-1. NLRP3 overexpression impaired the beneficial effect of FTZ by inducing pyroptosis, which was consistent with the findings from another research group [26].

MI triggers striking inflammatory responses, and hyperactivated inflammation plays a double-sword role in cardiac repair and regeneration. NLRP3 is a member of the inflammasome and can be activated upon tissue injury. It is connected with multiple forms of programmed cell death [27]. Thus, targeting NLRP3 and its downstream pathway to prevent pyroptosis has attracted significant attention [13, 28]. A recent study has shown that the NLRP3 inhibitor MCC950 can deregulate IL-1*β* [29] and can offer protection in a mouse model of MI [30] and even in larger animals [31]. Although, FTZ did not directly target NLRP3 in our network pharmacology analysis (Figure S7). In our study, IHC findings revealed that FTZ attenuated NLRP3 expression both *in vivo* and *in vitro* and reduced infarct size, which was partially reversed by the overexpression of NLRP3. The underlying mechanism of FTZ can be attributed to the inhibition of C-Caspase-1 and the premature release of IL-1*β* and IL-18, at least, partly by inhibiting pyroptosis. In spite of this, the exact molecular mechanism by which FTZ interferes with pyroptosis remains unclear. Collectively, our findings suggest that FTZ could attenuate the proinflammatory response and improve cardiac function.

As we know, reactive oxygen species, mitochondrial damage, infectious diseases, and episodes of MI could activate NLRP3 [32, 33]. Thus, we treated cardiomyocytes with H_2_O_2_ and found that FTZ treatment could mitigate the marker of pyroptosis genes. Several studies have demonstrated that fibroblasts are the primary cell type in pyroptosis and could serve as a target for the therapy. Therefore, we do not rule out the possibility of fibroblasts as targets of FTZ [34].

## 5. Conclusion

The primary goal of this study was to determine the effects of FTZ on MI in a mouse model and determine its potential mechanism of action, as these findings could have significant implications for the heart. A limitation with respect to the use of FTZ is the presence of complex components. FTZ treatment could inhibit cardiac ischemia-induced pyroptosis by downregulating NLRP3 and related cytokines including 1L-1*β* and 1L-18, which mitigate cardiac dysfunction. Our findings may promote the use of other traditional Chinese compounds in the management of heart diseases.

## Figures and Tables

**Table 1:** The herbal formula of FTZ.

English names	Scientific names of the herbs	Latin names of the medicinal materials	Chinese names	Materials
Citri Sarcodactylis Fructus	*Citrus medica L*.	*Citrus medica* linn. Var. sarcodactylis (noot.) swingle	Fo shou	Pulp
Fructus Ligustri Lucidi	*Ligustrum lucidum* W. T. Aiton	*Ligustrum lucidum* aiton	Nu zhen zi	Pulp
Salviae Miltiorrhizae Radix et Rhizoma	*Salvia miltiorrhiza* bunge	*Salvia miltiorrhiza* bunge	Dan shen	Rootstalk
Notoginseng Radix et Rhizoma	*Panax bipinnatifidus* var. *angustifoliu*s (Burkill) J. Wen	*Panax pseudoginseng* Wall. var. notoginseng (Burkill) Hoo and Tseng	San qi	Root
Coptidis Rhizoma	*Coptis chinensis* Franch	*Coptis chinensis* Franch	Huang lian	Rootstalk
Atractylodis Macrocephalae Rhizoma	*Atractylodes macrocephala* Koidz.	*Atractylodes macrocephala* Koidz	Bai zhu	Rootstalk
Cirsii Japonici Herba et Radix	*Cirsium japonicum* (Thunb.) Fisch. Ex DC	*Cirsium japonicum* DC	Da ji	Dry aerial parts or roots
Eucommiae Cortex	*Eucommia ulmoides* Oliv	*Eucommia ulmoides* Oliver	Du zhong	Bark

**Table 2:** Primer sequences used for PCR.

Genes		Primer sequences
NLRP3	Forward	GTGGAGATCCTAGGTTTCTCTG
	Reverse	CAGGATCTCATTCTCTTGGATC

GSDMD	Forward	CCATCGGCCTTTGAGAAAGTG
	Reverse	ACACATGAATAACGGGGTTTCC

Caspase-1	Forward	ACACGTCTTGCCCTCATTATCT
	Reverse	ATAACCTTGGGCTTGTCTTTCA

ASC	Forward	CTTGTCAGGGGATGAACTCA AAA
	Reverse	GCCATACGACTCCAGATAGT AGC

IL-1*β*	Forward	CCCTGCAGCTGGAGAGTGTGG
	Reverse	TGTGCTCTGCTTGAGAGGTGCT

IL-18	Forward	CATGTCAGAAGACTCTTGCGTCAA
	Reverse	GAGGGTCACAGCCAGTCCTCTT

*β*-actin	Forward	CCTGGCACCCAGCACAAT
	Reverse	GCTGATCCACATCTGCTGGAA

## Data Availability

The data used to support the findings of this study are included within the article.

## Abbreviations

FTZ: Fu Fang Zhen Zhu Tiao Zhi
C-Caspase-1: Cleaved caspase-l
IL: Interleukin
MI: Myocardial infarction
NLRP: NOD-like receptor protein
LAD: Left anterior descending
EF: Ejection fraction
FS: Fractional shortening
TUNEL: Terminal deoxynucleotidyl transferase dUTP nick end labeling
IHC: Immunohistochemical
IF: Immunofluorescence
H&E: Hematoxylin and eosin
ELISA: Enzyme-linked immunosorbent assay
LDH: Lactate dehydrogenase
NMVCs: Neonatal mice ventricular cardiomyocytes
LVIDd: Left ventricular internal diameter at end diastole
LVIDs: Left ventricular internal diameter at end systole
TTC: Triphenyltetrazolium chloride
DAPI: 4′,6-diamidino-2-phenylindole
RT-PCR: Reverse transcriptase-polymerase chain reaction.

## Supplementary Materials

Supplementary Materials Supplement Figure S1: HPLC chromatogram for FTZ. Supplement Figure S2 (a and b) and S4: identify the apoptotic cells type. Supplement Figure S3: dose-response study. Supplement Figure S5 (a and b): assessed the cardiomyocytes purity and NLRP3 plasmid transfection efficiency. Supplement Figure S6: inhibit NLRP3 expression and access cell viability. Supplement Figure S7: analyzing the pharmacology of networks.
