# Supplementary material for: Fu Fang Zhen Zhu Tiao Zhi Capsules Protect against Myocardial Ischemia by Inhibiting Cardiomyocyte Pyroptosis
Source: Evid Based Complement Alternat Med. 2022 Nov 2;2022:4752360. doi: 10.1155/2022/4752360 (PMC9646324; doi:10.1155/2022/4752360)

Identify the apoptotic cells type in vitro

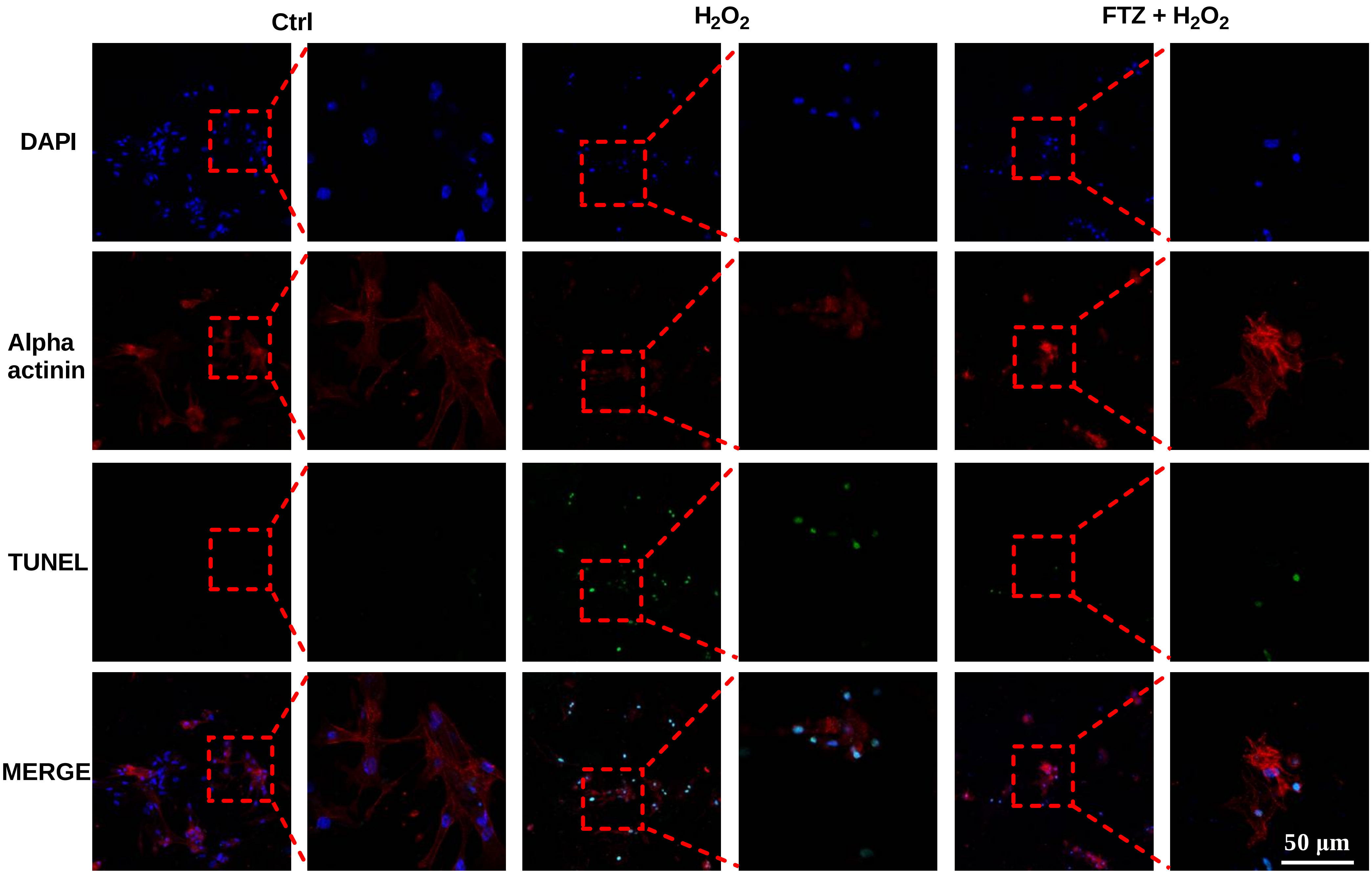

Supplement: Supplementary Materials — Supplement Figure S1: HPLC chromatogram for FTZ. Supplement Figure S2 (a and b) and S4: identify the apoptotic cells type. Supplement Figure S3: dose-response study. Supplement Figure S5 (a and b): assessed the cardiomyocytes purity and NLRP3 plasmid transfection efficiency. Supplement Figure S6: inhibit NLRP3 expression and access cell viability. Supplement Figure S7: analyzing the pharmacology of networks. [file 4752360.f1.zip › Figure S4-Identify the apoptotic cells type in vitro.pdf]
