# Supplementary figures and images for: Fu Fang Zhen Zhu Tiao Zhi Capsules Protect against Myocardial Ischemia by Inhibiting Cardiomyocyte Pyroptosis
Source: Evid Based Complement Alternat Med. 2022 Nov 2;2022:4752360. doi: 10.1155/2022/4752360 (PMC9646324; doi:10.1155/2022/4752360)

# Identify the apoptotic cells type in vivo

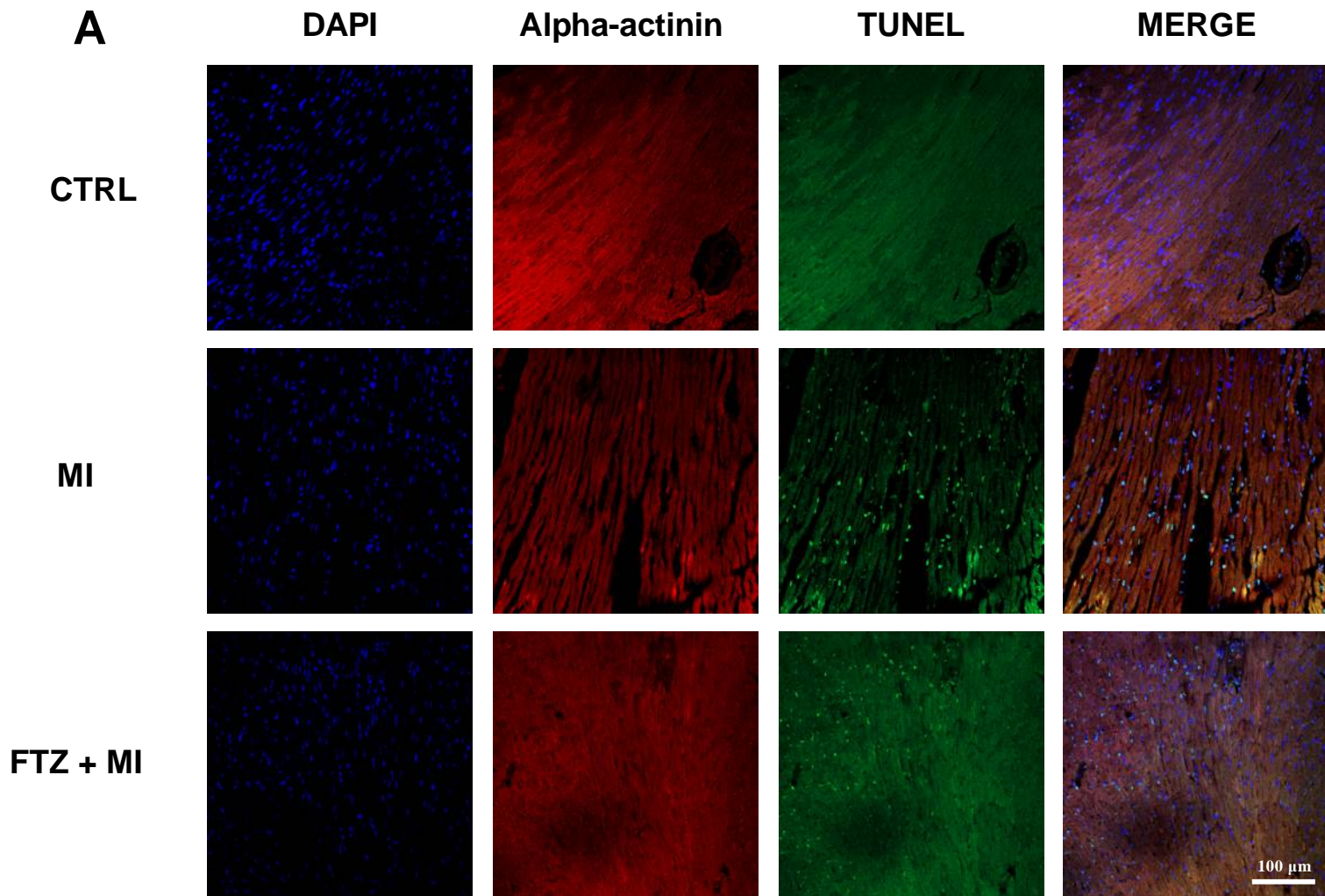

Supplement: Supplementary Materials — Supplement Figure S1: HPLC chromatogram for FTZ. Supplement Figure S2 (a and b) and S4: identify the apoptotic cells type. Supplement Figure S3: dose-response study. Supplement Figure S5 (a and b): assessed the cardiomyocytes purity and NLRP3 plasmid transfection efficiency. Supplement Figure S6: inhibit NLRP3 expression and access cell viability. Supplement Figure S7: analyzing the pharmacology of networks. [file 4752360.f1.zip › Figure S2A-Identify the apoptotic cells type in vivo.pdf]

# Analyze the cause of cell death

**B**

**DAPI**

**Alpha-actinin**

**NLRP3**

**MERGE**

**CTRL**

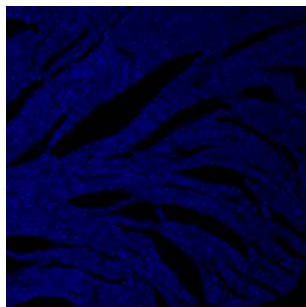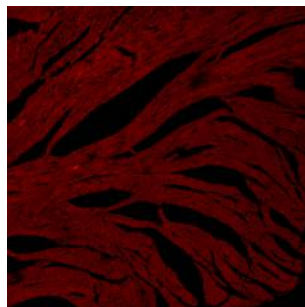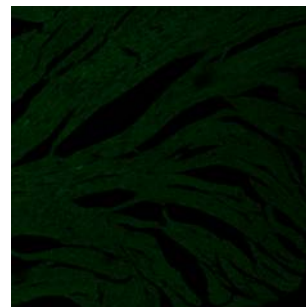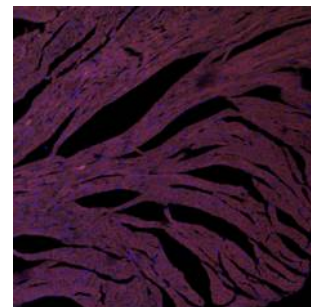

**MI**

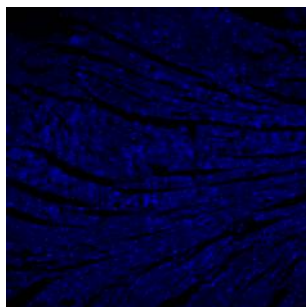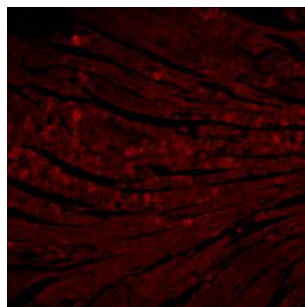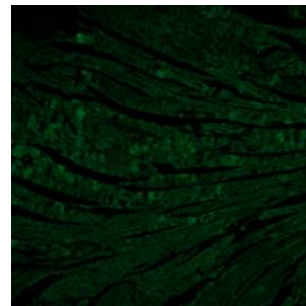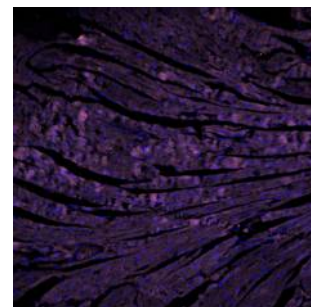

**FTZ + MI**

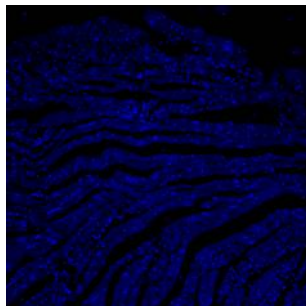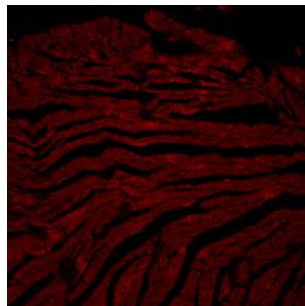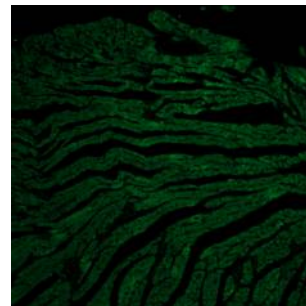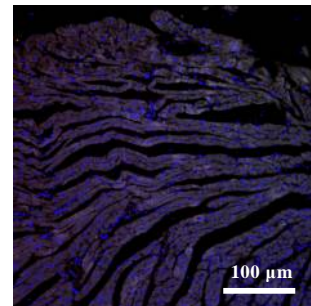

Supplement: Supplementary Materials — Supplement Figure S1: HPLC chromatogram for FTZ. Supplement Figure S2 (a and b) and S4: identify the apoptotic cells type. Supplement Figure S3: dose-response study. Supplement Figure S5 (a and b): assessed the cardiomyocytes purity and NLRP3 plasmid transfection efficiency. Supplement Figure S6: inhibit NLRP3 expression and access cell viability. Supplement Figure S7: analyzing the pharmacology of networks. [file 4752360.f1.zip › Figure S2B-Analyze the cause of cell death.pdf]

# Dose-response study of FTZ

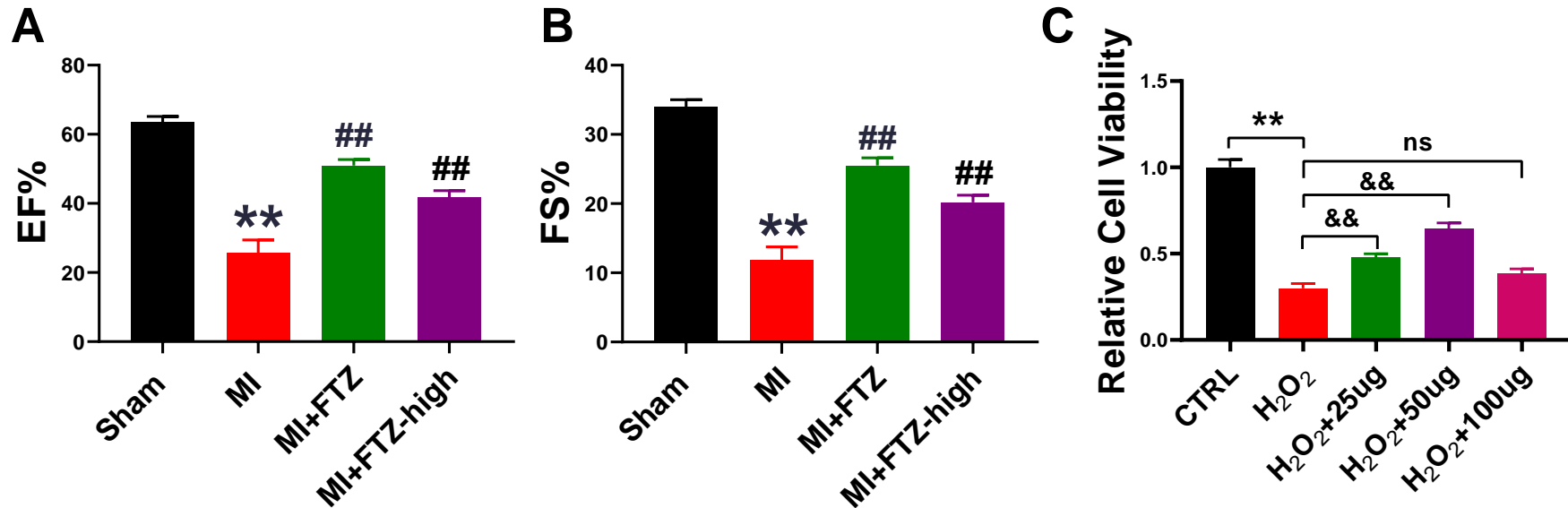

Supplement: Supplementary Materials — Supplement Figure S1: HPLC chromatogram for FTZ. Supplement Figure S2 (a and b) and S4: identify the apoptotic cells type. Supplement Figure S3: dose-response study. Supplement Figure S5 (a and b): assessed the cardiomyocytes purity and NLRP3 plasmid transfection efficiency. Supplement Figure S6: inhibit NLRP3 expression and access cell viability. Supplement Figure S7: analyzing the pharmacology of networks. [file 4752360.f1.zip › Figure S3-Dose-response study of FTZ.pdf]

Assessed the cardiomyocytes purity

**A**

**DAPI**

**Alpha-actinin**

**MERGE**

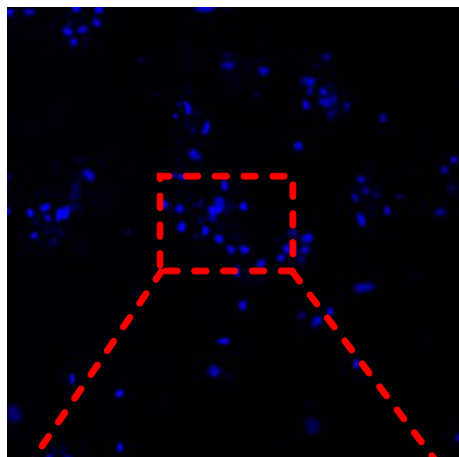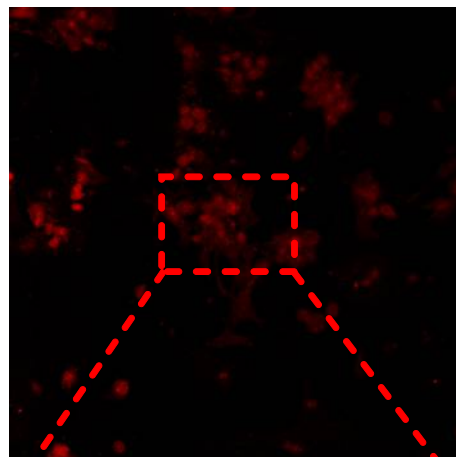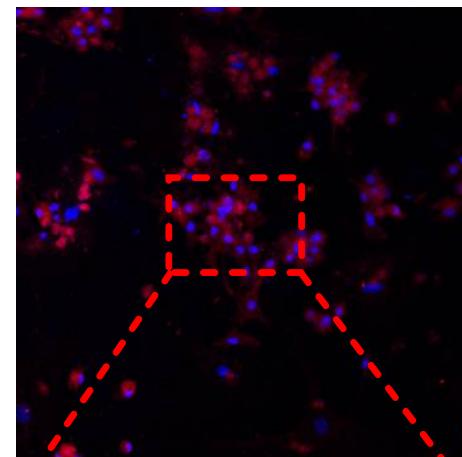

**Ctrl**

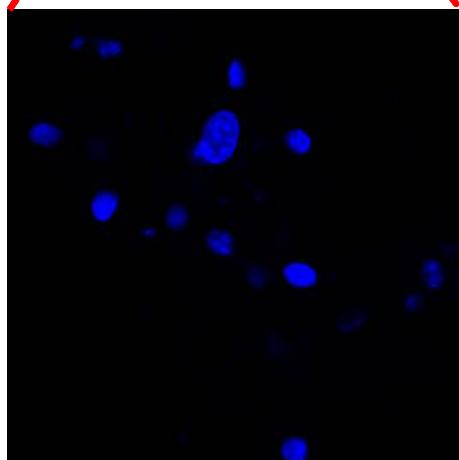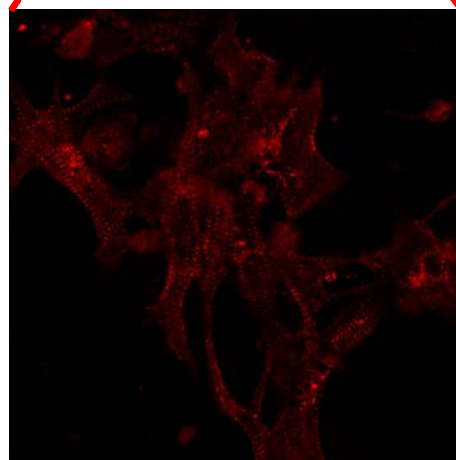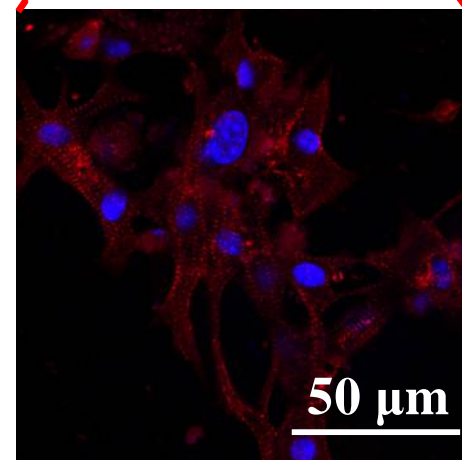

Supplement: Supplementary Materials — Supplement Figure S1: HPLC chromatogram for FTZ. Supplement Figure S2 (a and b) and S4: identify the apoptotic cells type. Supplement Figure S3: dose-response study. Supplement Figure S5 (a and b): assessed the cardiomyocytes purity and NLRP3 plasmid transfection efficiency. Supplement Figure S6: inhibit NLRP3 expression and access cell viability. Supplement Figure S7: analyzing the pharmacology of networks. [file 4752360.f1.zip › Figure S5A-Assessed the cardiomyocytes purity.pdf]

Assessed the NLRP3 plasmid transfection efficiency

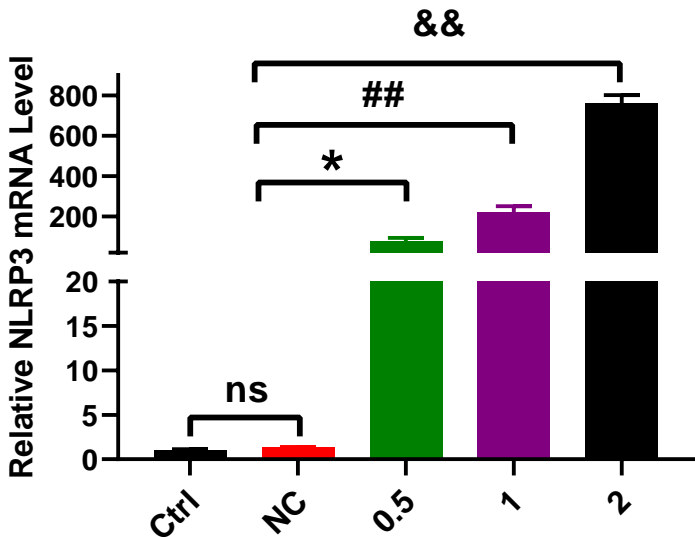

Supplement: Supplementary Materials — Supplement Figure S1: HPLC chromatogram for FTZ. Supplement Figure S2 (a and b) and S4: identify the apoptotic cells type. Supplement Figure S3: dose-response study. Supplement Figure S5 (a and b): assessed the cardiomyocytes purity and NLRP3 plasmid transfection efficiency. Supplement Figure S6: inhibit NLRP3 expression and access cell viability. Supplement Figure S7: analyzing the pharmacology of networks. [file 4752360.f1.zip › Figure S5B-Assessed the NLRP3 plasmid transfection efficiency.pdf]

# Inhibit NLRP3 expression and access cell viability

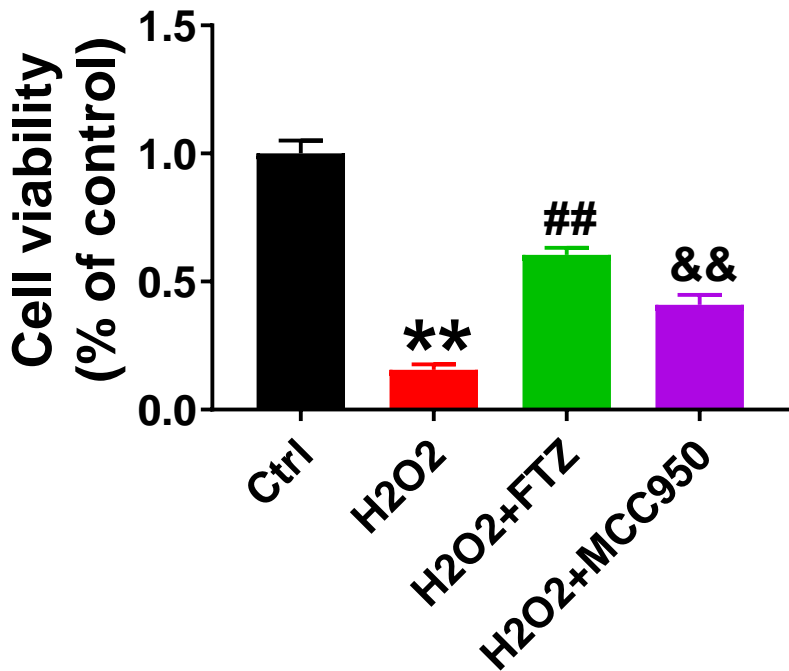

Supplement: Supplementary Materials — Supplement Figure S1: HPLC chromatogram for FTZ. Supplement Figure S2 (a and b) and S4: identify the apoptotic cells type. Supplement Figure S3: dose-response study. Supplement Figure S5 (a and b): assessed the cardiomyocytes purity and NLRP3 plasmid transfection efficiency. Supplement Figure S6: inhibit NLRP3 expression and access cell viability. Supplement Figure S7: analyzing the pharmacology of networks. [file 4752360.f1.zip › Figure S6-Inhibit NLRP3 expression and access cell viability.pdf]

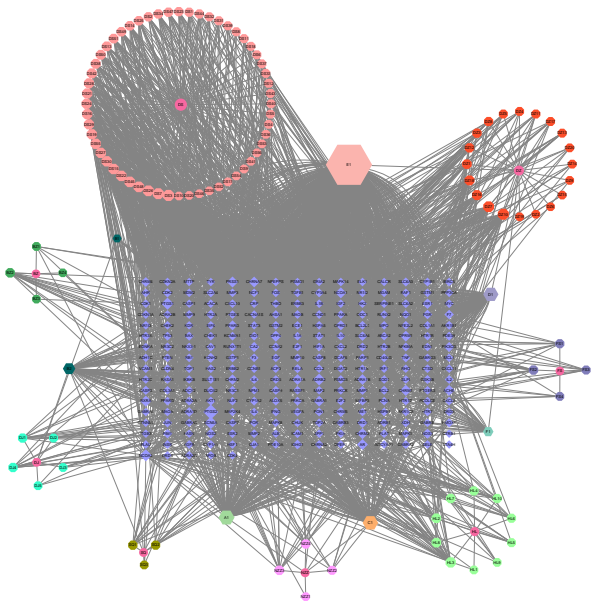

Supplement: Supplementary Materials — Supplement Figure S1: HPLC chromatogram for FTZ. Supplement Figure S2 (a and b) and S4: identify the apoptotic cells type. Supplement Figure S3: dose-response study. Supplement Figure S5 (a and b): assessed the cardiomyocytes purity and NLRP3 plasmid transfection efficiency. Supplement Figure S6: inhibit NLRP3 expression and access cell viability. Supplement Figure S7: analyzing the pharmacology of networks. [file 4752360.f1.zip › Figure S7-Analyzing the pharmacology of networks.pdf]

# Graphical abstract

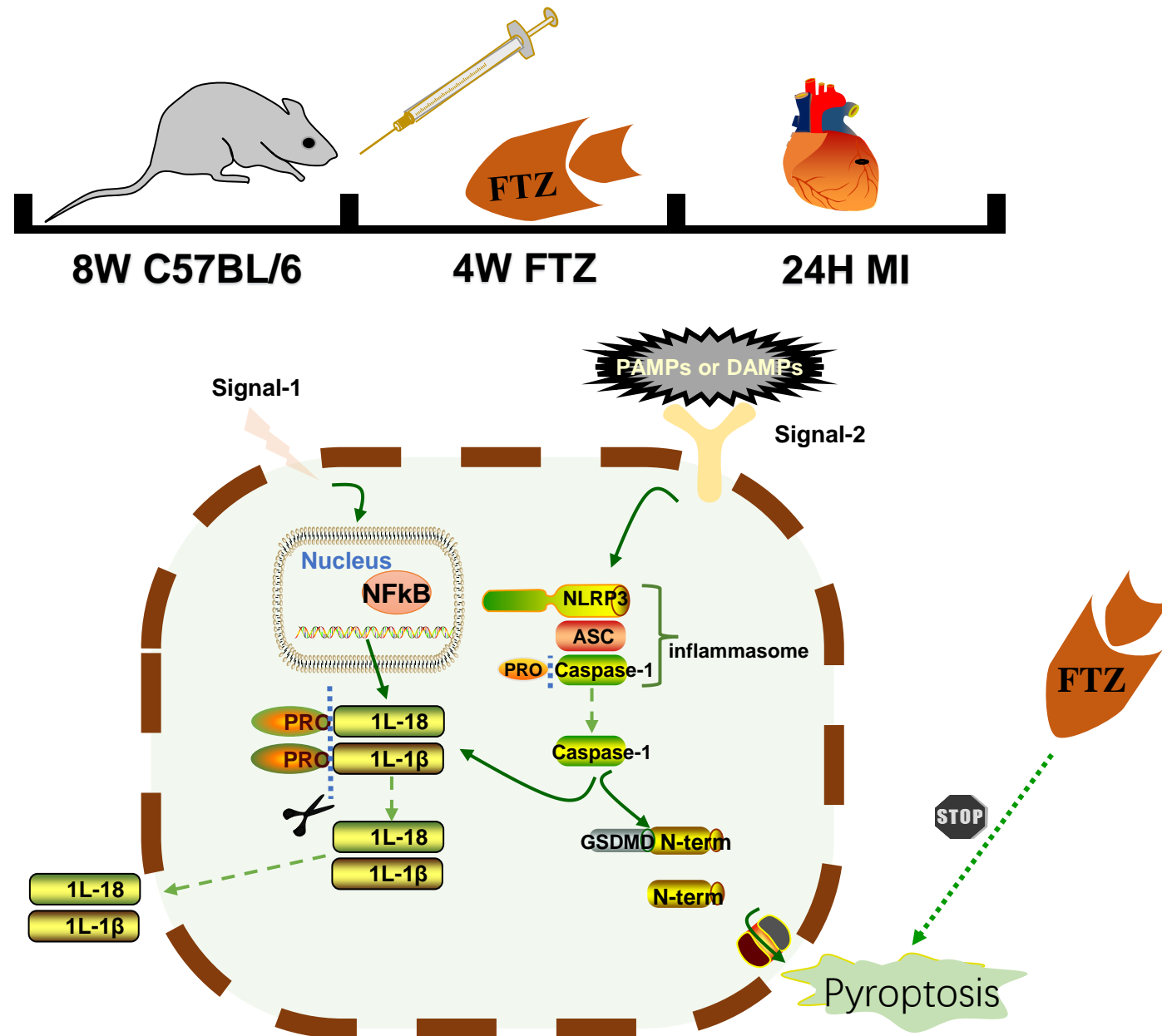

Supplement: Supplementary Materials — Supplement Figure S1: HPLC chromatogram for FTZ. Supplement Figure S2 (a and b) and S4: identify the apoptotic cells type. Supplement Figure S3: dose-response study. Supplement Figure S5 (a and b): assessed the cardiomyocytes purity and NLRP3 plasmid transfection efficiency. Supplement Figure S6: inhibit NLRP3 expression and access cell viability. Supplement Figure S7: analyzing the pharmacology of networks. [file 4752360.f1.zip › Graphical abstract.pdf]
